# Patterns of Child Mental Health Service Utilization Within a Multiple EBP System of Care

**DOI:** 10.1007/s10488-021-01179-7

**Published:** 2021-11-27

**Authors:** Joyce H. L. Lui, Lauren Brookman-Frazee, Alejandro L. Vázquez, Julia R. Cox, Debbie Innes-Gomberg, Kara Taguchi, Keri Pesanti, Anna S. Lau

**Affiliations:** 1 Department of Psychology, University of California, Los Angeles, Los Angeles, USA; 2 Department of Psychology, University of Maryland, College Park, USA; 3 Department of Psychiatry, University of California, San Diego, San Diego, USA; 4 Child and Adolescent Services Research Center, La Jolla, USA; 5 Department of Psychology, Utah State University, Logan, USA; 6 Los Angeles County Department of Mental Health, Los Angeles, USA

**Keywords:** Service utilization, EBP implementation, Child mental health, Disparities

## Abstract

The current study (1) characterizes patterns of mental health service utilization over 8 years among youth who received psychotherapy in the context of a community implementation of multiple evidence-based practices (EBPs), and (2) examined youth-, provider- and service-level predictors of service use patterns. Latent profile analyses were performed on 5,663,930 administrative claims data furnished by the county department of mental health. Multinomial logistic regression with Vermunt’s method was used to examine predictors of care patterns. Based on frequency, course, cost, and type of services, three distinct patterns of care were identified: (1) Standard EBP Care (86.3%), (2) Less EBP Care (8.5%), and (3) Repeated/Chronic Care (5.2%). Youth age, ethnicity, primary language, primary diagnosis and secondary diagnosis, provider language and provider type, and caregiver involvement and service setting were significant predictors of utilization patterns. Although the majority of youth received care aligned with common child EBP protocols, a significant portion of youth (13.7%) received no evidence-based care or repeated, costly episodes of care. Findings highlight opportunities to improve and optimize services, particularly for youth who are adolescents or transition-aged, Asian-American/Pacific Islander, Spanish-speaking, or presenting with comorbidities.

## Introduction

Nationally representative surveys estimate that 13% of children and adolescents suffer from a mental health disorder in the U.S., but only a third to a half of these youth receive services (Merikangas et al., 2010, 2011). In a recent meta-analysis, Duong et al. found that outpatient clinics and schools were the most accessed settings for mental health services, although service rates in these settings remain below 45% among youth with elevated symptoms or diagnoses and many youth remain unserved (Duong et al., 2020). Furthermore, among youth who initiate services, evidence suggests that the continuity of that care is poor. For example, Saloner et al. (2014) found that only 33% of youth received minimally adequate care, defined as four or more total visits with a mental health professional. A recent report from Mental Health America found that only 27% of youth with severe depression accessed more than seven visits total in the past year (Mental Health America, 2020). Such sobering figures highlight the continued need to address mental health care gaps among children and adolescents.

Evidence-based practices, or EBPs, are increasingly implemented across publicly funded mental health services for youth as policymakers and payors strive for quality improvement. Many EBPs exist for common presenting concerns for youth such as depression (Weersing et al., 2017), anxiety (Higa-McMillan et al., 2016), trauma (Dorsey et al., 2017), and disruptive behaviors (e.g., Kaminski & Claussen, 2017; McCart & Sheidow, 2016). EBPs are typically designed to be time-limited, focused treatment, often ranging from 12 to 20 sessions. In a study examining the compatibility of EBPs in the context of community mental health, Whiteside et al. (2020) used 13 sessions as a benchmark to assess whether youth received a sufficient dose of evidence-based care in a single year based on average number of sessions in common child treatment manuals and previous efficacy trials. However, there is limited empirical data on the minimum treatment length required for improved outcomes and mixed results on a dose response relationship for youth receiving care in community mental health (Bickman et al., 2002; Kirk et al., 2019).

There is surprisingly limited research on mental health service utilization patterns among youth and this has been identified as a barrier to policy reforms to improve quality of care (Saloner et al., 2014). The research that has been conducted to date has examined youth mental health service use based on number of sessions attended and overall duration of treatment (e.g., Saloner et al., 2014), and does not tell us *how* youth engage with mental health services. For example, it is unclear if care is episodic, chronic, continuous, or sporadic in course and at what intensity care is delivered. Empirical approaches such as latent class or latent profile analyses can allow us to explore patterns of use related to course and intensity. Past efforts on understanding mental health service utilization patterns have been limited to specific subpopulations (e.g., immigrants, refugees, specific diagnostic groups; Anderson et al., 2013; Derr, 2016; Olfson et al., 2003; Pastor et al., 2017; Salami et al., 2019; Wu et al., 2001). An exception is work by Reid et al. in Ontario, Canada. In two studies (2011, 2019), these authors identified five distinct patterns of care among youth receiving mental health services across 4–5 years. They found significant variability in the course and intensity of services, such that the majority of children (53%) received minimal care, characterized by infrequent visits over the course of a few months, while 23% of youth received prolonged periods of care for more than 2 years. About 20% of youth received a single discrete/acute episode of care with regular visits over 9.6 months. There was also a pattern of episodic care (8%), characterized by infrequent visits across multiple episodes of care spanning 3.5 years. The final two patterns were intensive (13%) and ongoing/intensive-episodic (6%), characterized by high volume of visits over multiple years. This pioneering work challenges the field to consider how common patterns of service receipt align with treatment protocols as tested in effectiveness trials and alternative models of care delivery that address a range of levels of need (e.g., triage or stepped-care models).

A major gap in the youth mental health services literature is the lack of data on the delivery of EBPs and associated patterns of psychotherapy service utilization in routine care settings. Several county- and state-wide EBP initiatives exist including in Los Angeles County (Regan et al., 2017), New York State (Acri et al., 2019), and Washington State (Stoner, 2018). Despite increasing implementation of EBPs, we know little about youth service utilization patterns within community EBP implementation efforts and what factors may affect patterns of service use in these contexts. Triplett et al. (2021) surveyed 376 therapists who participated in a state-funded EBP training initiative in Washington State about their delivery of EBPs following completion of training and consultation. When asked to report on their delivery of an EBP protocol for their most “complete” case, therapists reported delivering 12.4 sessions on average. Furthermore, whereas 20.1% of therapists reported not having completed the EBP with the client, 21.2% of therapists reported that the client’s treatment discontinued after completing the EBP, and 58.7% of therapists reported continuing therapy (with or without continued delivery of EBP elements). These findings suggest that service utilization patterns may influence EBP delivery in routine care. More research is needed to understand the range of patterns of service utilization in a context of multiple EBPs implementation in routine mental health care systems and factors associated with more and less optimal patterns of care.

Drawing on Anderson’s behavioral model of health service utilization (Andersen & Davidson, 2007), several predictors at the youth, parent, and provider level have been identified to be associated with patterns of mental health service use. Research reveals racial/ethnic disparities in mental health service utilization; youth from racial/ethnic minority groups (e.g., Black, Latinx, Asian) are less likely to enter mental health services (Garland et al., 2005; Le Cook et al., 2013; Saloner et al., 2014) and more likely to drop-out of care prematurely (de Haan et al., 2018) relative to non-Hispanic Whites. Youth’s gender is also associated with service use, such that caregivers are more likely to seek care for boys than girls (Merikangas et al., 2010; Reid et al., 2019; Saloner et al., 2014). Scholarship focusing on the effect of youth’s age is limited to older studies and show mixed results (Edlund et al., 2002; Hazen et al., 2004; Stein et al., 2013). Unsurprisingly, perceptions of need for treatment and the presence of comorbidity and impairment are associated with increased service use (Chavira et al., 2009; Reid et al., 2019; Ryan et al., 2015); however, to our knowledge, no research to date has examined differential patterns of service use by youth diagnosis. Caregiver involvement in youth therapy is critical in most EBPs for externalizing behaviors (Kaminski & Claussen, 2017) and has been associated with larger treatment effects in cognitive behavioral therapy for youth internalizing behaviors as well (Sun et al., 2019). In community service contexts serving high proportions of racial/ethnic minority families, lower caregiver engagement has been found when therapists are unable to deliver services in non-English languages (Barnett et al., 2019). Ultimately, identifying predictors of mental health service use patterns can inform strategies for improving treatment access and quality.

### The Current Study

There is a dearth of research on youth’s service utilization patterns in community mental health systems implementing EBPs. Past research has focused on describing the types or rates of services received (i.e., number of sessions attended), focused on subpopulations (i.e., a particular diagnosis or demographic group), with brief study duration, and in systems where no EBPs are implemented. The current study extends the children’s mental health services and implementation research literature by (1) characterizing patterns of mental health service utilization among youth receiving psychotherapy using latent profile analyses within a system employing multiple EBPs, (2) focusing on the largest county public mental health service system in the U.S. serving youth with a variety of common presenting concerns, and (3) exploring patterns of care over 8 years of a community EBP implementation initiative. To the extent that the EBP implementation initiative supported the delivery of structured, high-quality care, we may expect to see evidence of service use pattern aligned with these parameters. Furthermore, identifying factors associated with distinct patterns of utilization may inform how to optimize delivery of care within community EBP implementation efforts.

## Method

### Study Context

Data were collected as part of the Knowledge Exchange on Evidence-Based Practice Sustainment (4KEEPS) study [R01MH100134; MPIs: Lau and Brookman-Frazee (2015)], which examined the sustainment of multiple child EBPs within a system-driven implementation by the Los Angeles County Department of Mental Health (LACDMH, 2016). LACDMH is the largest county public mental health system in the United States and serves more than 250,000 individuals yearly (LACDMH, 2016). As part of the Prevention and Early Intervention (PEI) initiative that began in 2010, LACDMH implemented a fee-for-service reimbursement of EBPs for children and youth across all of their agencies (see LACDMH, 2016 for details about PEI initiative). PEI is intended for youth early in their course of illness who can benefit from brief, time-limited EBPs. Providers were required to deliver approved EBPs (e.g., Trauma Focused Cognitive Behavior Therapy, Parent–Child Interaction Therapy, Triple P, etc.) in order to receive reimbursement to PEI-allocated funds within this model. Other funding mechanisms (e.g., County General Funds, Medicaid) do not impose requirements on EBP delivery for reimbursements. The specific EBP delivered was specified and linked to a procedure code into the billing process. LACDMH specified the expected frequency and treatment length for each EBP (see Table 1 for a list of the most frequently used EBPs in the county) and the recommended maximum treatment episode duration is 18 months. The system-driven implementation of EBPs provides a unique opportunity to characterize mental health service utilization for an entire population served within a single public health system and to examine how patterns of care align with expected EBP course of treatment in an implementation as usual context.

**Table 1:** Frequency and treatment length of common EBPs in LACDMH

Evidence-based practice (EBP)	Frequency	Treatment length
Managing and Adapting Practice (MAP)	Not specified	Max ~ 12 months
Trauma-Focused Cognitive Behavioral Therapy (TF-CBT)	1 × per week	12–16 sessions
Seeking Safety	1 × per week	25–50 sessions
Positive Parenting Program	1 × x per week	10 sessions (individual) 5 sessions (group)
Child-Parent Psychotherapy	1 × per week	50 weeks
Interpersonal Psychotherapy for Depression	1 × per week	8–20 sessions
Parent–Child Interaction Therapy (PCIT)	1 × per week	16–24 sessions

*Source* LACDMH (2016)

### Procedure

This study utilized administrative claims data furnished by LACDMH. All available outpatient claims to PEI funds for Fiscal Year 2009/2010 to Fiscal Year 2017/2018 for children and transition-aged youth (≤ 25 years old) were extracted (*n* = 6, 914,533 claims). Because the goal of the study was to characterize patterns of mental health service utilization among youth receiving psychotherapy, only youth who had at least one psychotherapy claim were included in analyses. Youth who exclusively received other services (e.g., medical management only) were omitted (*n* = 20,462; 17%). To ensure that each youth had at least one year opportunity to receive services during the study period, we removed youth whose first claim was initiated in the last fiscal year. The final sample included 5,663,930 claims for 138,359 youth, submitted by 13,067 treatment providers. Characteristics of the youth and therapist samples are found in “Results” below. Study procedures were approved by Institutional Review Boards at [University of California San Diego, University of California Los Angeles, and Los Angeles County Department of Mental Health].

#### Service Use Indicators

Eight indicators were derived from claims data to characterize the pattern of service utilization for each youth. Each claim was associated with the date of service, procedure code (e.g., individual psychotherapy, group psychotherapy, family therapy, collateral, medication management, crisis management, evaluation/assessment, etc.), EBP code (e.g., MAP, TF-CBT, no/unknown EBP), provider language, provider discipline/type, service setting (e.g., office, home, school, etc.), youth demographics (e.g., age, gender, race/ethnicity, primary diagnosis, language), and cost of service. Service use indicators were developed to characterize the volume, continuity/frequency, duration, EBP receipt, and cost of services.

##### Volume

The total volume of claims was indexed by total psychotherapy claims and total non-psychotherapy claims. Total psychotherapy claims (TotPsych) were calculated by summing counts of all psychotherapy (e.g., individual, group, family therapy) claims per youth. Total non-psychotherapy claims (TotNonPsych) were the sum of all other claims (e.g., medication management, case management, crisis management, evaluation/assessment) per youth.

##### Continuity

Continuity was indexed in two ways. First, the average number of days between psychotherapy claims was calculated per youth (GapMean). Second, episode count was indexed by total number of episodes (TotEpisode), with a new episode defined when there was 180 or more days between consecutive psychotherapy claims per youth-provider dyad.

##### Duration

Duration of service was calculated as the number of active months each youth had a psychotherapy claim (ActiveMonth).

##### EBP Receipt

EBP Receipt was indexed by the number of unique EBPs received and the percentage of psychotherapy claims not tied to an EBP. The number of EBPs was the count of total unique EBPs received by a youth (EBPCount). The percentage of no EBP was calculated as the percentage of psychotherapy claims tied to a no/unknown EBP code (%NoEBP).

##### Cost

Total cost was calculated as the sum of costs associated with all claims per youth.

#### Service Use Predictors

Youth demographics, provider demographics, and service characteristics were included as predictors.

##### Youth Demographics

The following youth demographic variables associated with youth’s first claim were used: age, gender, race/ethnicity, language, primary diagnosis, secondary diagnosis, and year of first claim. Age was categorized as childhood (0–12), adolescence (13–17), and transition-aged (18–25). Race/ethnicity was categorized as non-Hispanic White, Hispanic, Black/African American, Asian American/Pacific Islander, Another Minoritized Ethnicity, and Not Reported. Youth’s preferred primary language was dichotomized into Spanish or English. Primary diagnosis was categorized into Internalizing Disorders (e.g., anxiety disorders, mood disorders), Externalizing Disorders (e.g., attention-deficit/hyperactivity disorder, disruptive behavior disorders), Trauma or Stress-Related Disorders (e.g., post-traumatic stress disorder, adjustment disorders), and Other Disorders. Because the majority of youth did not have a secondary diagnosis (70.9%), secondary diagnosis was dichotomized into a binary variable (yes/no). Year of first claim was included as a covariate to account for youth’s varying opportunity for ensuing service utilization during the study period.

##### Provider Demographics

The following provider demographics represent information associated with the youth’s first claim. Provider discipline/type was dichotomized into mental health therapists (i.e., Marriage and Family Therapists, Social Workers, Counselors, Psychologists, Trainees) versus other providers (i.e., Psychiatrist, Nurse, Rehabilitation Practitioner, Occupational Therapist, Other). Language, or language(s) that providers reported they can deliver services in, was dichotomized into English-only and Language(s) other than English.

##### Service Characteristics

Service setting for the care provided was dichotomized into Office Only or Community (i.e., a youth had at least some claims from a community setting such as home or school). The percentage of caregiver involved claims was calculated per youth and categorized as 0 (less than 50% caregiver involvement) and 1 (50% or more caregiver involvement). The following types of psychotherapy procedural codes indexed caregiver involvement: collateral, family therapy, family therapy with client, and multifamily group therapy.

### Data Analytic Plan

There was no to minimal missing data for the majority of variables (no missing data for procedure code, service setting, service date, cost, or youth’s age; < 3% missing for therapist language and discipline and youth’s gender, diagnosis, and primary language, and EBP code). Missing EBP code (*n* = 6233 claims) was classified as “no EBP”, missing youth’s ethnicity (*n* = 21,406; 13.9% of youth) was classified as “Ethnicity not reported,” and missing youth’s secondary diagnosis (*n* = 98,093; 70.9% of youth) was classified as “No Secondary Diagnosis.”

Latent profile analyses (LPA) were conducted to determine underlying subgroups with different patterns of care using eight indicators related to Volume, Continuity, Duration, EBP Receipt, and Cost. LPA was particularly suited because it does not assume that the population sampled is homogenous, does not assume a priori the number of groups/profiles, and enables the identification of patterns where many continuous variables are considered simultaneously (Stanley et al., 2017). LPA was conducted using full maximum likelihood estimation using Mplus 8 (Muthén & Muthén, 2017). Models were built successively with increasing number of profiles beginning with 1, and model fit was assessed. Models were tested on the full sample as well as five subsamples randomly drawing 15% of the sample (*n* ~ 20,753 youth per subsample) to examine the robustness of LPA solutions. Although no common standard criteria exist for LPA model selection, several guidelines and rules of thumb have been suggested. The following criteria were used to determine the optimal number of profiles to retain: (1) lower Bayesian information criterion (BIC) and sampled-adjusted Bayesian information criterion (SABIC), (2) a statistically significant bootstrapped likelihood ratio test (BLRT; indicating the additional profile resulted in improved model fit), (3) high posterior probabilities for each profile (≥ 0.70), (4) no less than 1% of sample in a given profile, and (5) interpretability of profiles, theoretical meaningfulness, and parsimony (Jung & Wickrama, 2008; Marsh et al., 2009; Nylund et al., 2007; Stanley et al., 2017; Tein et al., 2013). Due to computational demands required to conduct the BLRT, random draws of 1% of the sample (*n* ~ 1540 individuals per subsample) was selected to run the BLRT and this process was iterated five times for each LPA model. For interpretation of the best-fitting model, indicator scores were z-scored, with scores above + 0.5 labeled as high, scores less than − 0.5 labeled as low, and scores in between labeled as average (Ekblom-Bak et al., 2020).

To examine predictors of care patterns/profile membership, Vermunt’s method was used. Collier and Leite (2017)’s Monte Carlo simulation found that Vermunt’s method, a type of three-step estimation approach, yielded more accurate results for predictors of profile membership than single-step and other three-step approaches. Vermunt’s method first estimates profile membership using only indicator variables, then estimates the most likely profile membership using posterior probabilities, and finally regresses profile membership on predictor variables while accounting for measurement error (Vermunt, 2010). Eleven predictor variables were entered as auxiliary variables with the R3STEP command in Mplus (Asparouhov & Muthén, 2014). Listwise deletion removed 1888 youth for the prediction model resulting in a sample of 136,471 youth (98.6%). Chi-square tests indicated statistically significant differences on the predictor variables between the retained and excluded sample of youth, however, effect sizes were close to 0 (range − 0.01 to 0.09). Given the sensitivity of chi-square test and small effect sizes of differences between retained and excluded youth, data were treated as Missing Completely at Random (MCAR). Listwise deletion of missing data is acceptable under the MCAR mechanism (Enders, 2010).

## Results

### Youth and Service Characteristics

Sample characteristics are presented in Table 2. The youth sample (*n* = 138,359) consisted of slightly more males (54.6%) then females (45.4%) with a mean age of 11.82 years (SD = 4.79 years). Youth were predominantly from an ethnic minoritized group (85.7%), with the largest representation of Hispanic/Latinx youth (67.4%). Youth’s primary diagnoses varied across common mental health concerns seen in community mental health, including Mood Disorders (19.6%), Trauma and Stress-Related Disorders (19.6%), Disruptive Behavior Disorders (19.1%), and Anxiety Disorders (12%). The majority of youth did not have a secondary diagnosis (70.9%). Youth were served by providers (*n* = 13,067) representing multiple disciplines, with social workers and marriage and family therapists comprising 49.2% of providers. A sizable portion of providers were classified as providing services in a language other than English (29.9%).

**Table 2:** Sample characteristics

	*N* (%) or Mean (*SD*)
Child Level (*n* = 138,359)	
Child Age	11.82 (4.79)
Child Gender	
Male	75,539 (54.6%)
Female	62,795 (45.4%)
Child Race/Ethnicity	
Hispanic/Latinx	93,308 (67.4%)
African American	20,025 (14.5%)
Non-Hispanic White	10,424 (7.5%)
Asian American/Pacific Islander	2287 (1.7%)
Another Minoritized Ethnicity^a^	2904 (2.1%)
Not Reported	9411 (6.8%)
Child Primary Diagnosis	
Mood	42,529 (19.6%)
Anxiety	16,534 (12%)
Trauma and Stress	27,093 (19.6%)
Disruptive Behavior	26,470 (19.1%)
ADHD	12,811 (9.3%)
Other	12,909 (9.3%)
Child Secondary Diagnosis	
Mood	4540 (3.3%)
Anxiety	3487 (2.5%)
Trauma and Stress	6346 (4.6%)
Disruptive Behavior	3933 (2.8%)
ADHD	3402 (2.5%)
Other	18,558 (13.4%)
No Secondary Diagnosis	98,093 (70.9%)
Child Primary Language	
English	99,313 (71.8%)
Spanish	37,126 (26.8%)
Another Language	1430 (1%)
Provider Level (*n* = 13,067)	
Provider Type/Discipline^b^	
Social Work/Counselor	3596 (27.5%)
Marriage and Family Therapy	2837 (21.7%)
Psychologist	454 (3.5%)
Trainee	1393 (10.7%)
Rehabilitation/Occupational Professional	2716 (20.8%)
Medical/Psychiatry	1002 (7.7%)
Case Manager	732 (5.6%)
Provider Language	
English Only	9024 (61.4%)
Spanish	3912 (29.9%)
Another Language	1016 (7.8%)
Claims Level (*n* = 5,663,930)	
Procedure	
Psychotherapy	4,124,960 (72.8%)
Medical Management	449,747 (7.9%)
Case Management	249,973 (4.4%)
Evaluation and Assessment	248,866 (4.4%)
Crisis Management	19,265 (.3%)
Other or Not Reported	571,119 (10.1%)
Service Setting	
Office	3,727,825 (65.8%)
Home	1,043,666 (18.4%)
School	892,439 (15.8%)
EBP (Psychotherapy claims only *n* = 4,124,960)	
Specified EBP	3,798,179 (92.1%)
No or Unknown EBP	326,781 (7.9%)
Caregiver Involvement Specifier	1,690,231 (40.9%)

Child level variables represent claims aggregated to the child level. Provider level variables represent claims aggregated to the provider level. Claims level variables represent raw claims variables

^a^Another Minoritized Ethnicity includes American Natives (.3%) and individuals categorized as “Other Non-White” and “Other” (1.8%) in the administrative claims data

^b^MFT, SW, Psychologist, Case Managers, and Trainees were coded as Therapists; Rehab/Occupational Professional and Medical/Psychiatry were coded as Other Providers

The majority of mental health service claims were psychotherapy claims (72.8%). Of those, 40.9% of claims involved a caregiver or family members. Services were primarily provided in an office setting (65.8%). The majority of psychotherapy claims were associated with a specified EBP (92.1%) and 7.9% of psychotherapy claims were not.

### Aim 1: Patterns of Service Utilization

**Fig. 1:**
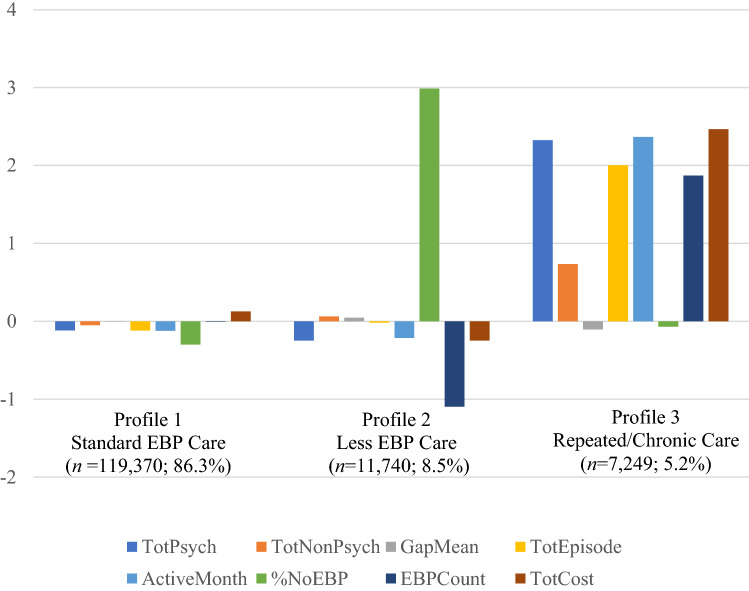
Mental health service utilization profiles. Figure illustrate z-scores on the following indicators: Total psychotherapy claims (TotPsych), total non-psychotherapy claims (TotNonPsych), average days between psychotherapy claims (GapMean), total number of psychotherapy episodes (TotEpisode), number of psychotherapy active months (ActiveMonth), percentage of psychotherapy claims with no EBP (%NoEBP), number of specified EBPs received (EBPCount), and total cost (TotCost). Scores above + 0.5 are considered high, scores less than − 0.5 are considered low, and scores in between are considered average (Ekblom-Bak et al., 2020)

LPA models specifying 1 to 4 profiles were conducted. Fit indices for LPA models are presented in Table 3. BIC and SABIC values decreased with each additional profile and the BLRT indicated each additional profile significantly improved fit. However, the four-profile solution yielded profiles represented by less than 1% of the sample, limiting its utility. Thus, considering all fit criteria and interpretability, the three-profile model was retained. The three profiles are presented in Fig. 1 and means on indicators across profiles are presented in Table 4.

**Table 3:** Fit indices for LPA models

# of Profiles	Loglikelihood	AIC	BIC	SABIC	Entropy	BLRT Loglikelihood	BLRT *p* value	Posterior Probabilities	Profile Membership Distribution (%)
1	− 3,838,313.18	7,676,658.35	7,676,815.75	7,676,764.90	–	–	–	–	–
2	− 3,716,508.84	7,433,067.67	7,433,313.61	7,433,234.16	0.981	− 35,488.31	< 0.001		
								0.951	5.19
								0.997	94.81
3	− 3,622,817.07	7,245,702.14	7,246,036.62	7,245,928.57	0.983	− 33,849.48	< 0.001		
								0.985	8.44
								0.995	86.26
								0.949	5.30
4	− 3,576,936.87	7,153,959.74	7,154,382.76	7,154,245.11	0.985	− 32,865.53	< 0.001		
								0.948	5.54
								0.984	8.27
								0.995	85.40
								0.971	0.80

*AIC* akaike information criteria, *BIC* Bayesian information criteria, *SABIC* sample-adjusted Bayesian information criterion, *BLRT* bootstrapped likelihood ratio test. A value of *p* < 0.05 indicates that a k − 1 class model provides better fit than a k-class model. The profile membership distribution indicates the percentage of the sample in each profile. LPA models were conducted on the full sample (*n* = 138,359) as well as five subsamples with 15% of youth (*n* = 20,754). The subsample analyses were conducted to test the robustness of the LPA solutions. Across subsamples, the 3-profile solution was the most robust

**Table 4:** Means of indicators for the three service utilization profiles

		Profile 1			Profile 2			Profile 3		
		Standard EBP Care			Less EBP Care			Repeated/Chronic Care		
		(*n* = 119,370; 86.3%)			(*n* = 11,740; 8.5%)			(*n* = 7249; 5.2%)		
	Variables	Mean	Median	SD	Mean	Median	SD	Mean	Median	SD
Volume	TotPsych	24.98	20.0	20.97	19.52	7.0	74.64	126.13	107.0	81.54
	TotNonPsych	7.91	5.0	12.92	15.17	3.0	175.65	57.42	32.0	143.99
Continuity	GapMean	12.01	7.5	22.02	13.0	6.53	33.17	9.58	7.91	6.98
	TotEpisode	1.72	1.0	1.13	1.94	1.0	3.95	6.36	5.0	4.96
Duration	ActiveMonth	7.85	7.0	5.68	6.81	3.0	21.59	35.86	31.0	19.5
EBP receipt	%NoEBP	3.14	0	8.39	84.39	100	19.75	8.57	1.49	14.31
	EBPCount	1.23	1.0	0.48	0.54	0	0.66	2.4	2.0	0.94
Cost	TotCost	6460.87	5114.65	5165.46	5301.2	2520.02	16,383.39	30,925.86	27,405.15	15,928.42

#### Profile 1: Standard EBP Care

This profile was the largest of the sample (86.3%) and had average levels across all indicators. Youth on average received 24.98 number of psychotherapy sessions across 7.85 months, with 12.01 days between sessions. On average, only 3.14% of psychotherapy claims were not associated with EBP delivery. Examining the ratio of psychotherapy claims to non-psychotherapy claims suggest that youth in this profile primarily received psychotherapy services. Youth had on average 1.72 discrete episodes of care. Average cost per youth was $6460.87.

#### Profile 2: Less EBP Care

This profile comprised 8.5% of youth. Youth represented in this profile received on average 19.52 psychotherapy sessions across 6.81 months, with 13 days between sessions. These sessions are primarily not associated with EBP delivery (*M* = 84.39% of psychotherapy claims were no EBP claims). Youth in this profile received roughly comparable numbers psychotherapy and non-psychotherapy claims and had on average 1.94 discrete episodes of care. Average cost per youth was $5301.20.

#### Profile 3: Repeated/Chronic Care

This profile represented a small proportion of youth (5.2%). Youth on average received 126.13 sessions of psychotherapy across 35.86 months, with 9.58 days between sessions. They received multiple specified EBPs (*M* = 2.4), had a high number of psychotherapy claims (*M* = 126.13), and other types of mental health services (*M* = 57.42), and had high numbers of discrete episodes of care (*M* = 6.36). Average cost per youth was $30,925.86.

### Aim 2: Predictors of Service Utilization Patterns

Results from the multinomial logistic regression using Vermunt’s method revealed several significant predictors on profile membership. Profile 1, Standard EBP Care, was used as the reference group. Thus, a significant predictor indicated higher or lower risk of being in one of the other profiles relative to the Standard EBP Care group. See Table 5 for results.

**Table 5:** Multinomial logistic regression of predictor variables on membership in service utilization patterns

	Profile 2 Less EBP Care			Profile 3 Repeated/Chronic Care		
Predictor	Log odds	Risk ratio	*p*	Log odds	Risk ratio	*p*
Child Age (ref: Child)						
Adolescent	0.15	1.16	< 0.001*	0.09	1.09	0.004*
Transition-Aged Youth	0.73	2.07	< 0.001*	0.64	1.90	< 0.001 *
Child Gender (ref: Female)	0.02	1.02	0.31	0.14	1.15	< 0.001*
Child Language Spanish (ref: English)	0.08	1.08	0.002*	0.00	1.00	0.98
Child Ethnicity (ref: Hispanic)						
Non-Hispanic White	0.06	1.06	0.17	0.22	1.25	< 0.001*
Black	0.06	1.06	0.07	0.03	1.03	0.48
Asian American/Pacific Islander	0.25	1.28	0.001*	0.10	1.10	0.35
Another Minority Not Listed	0.08	1.09	0.25	0.39	1.47	< 0.001*
Not Reported	0.89	2.43	< 0.001*	0.08	1.08	0.46
Child Primary Diagnosis (ref: Internalizing)						
Externalizing	− 0.07	0.93	0.01*	0.41	1.51	< 0.001*
Trauma and Stress-Related	− 0.21	0.81	< 0.001*	0.32	1.38	< 0.001*
Other	0.00	1.00	0.92	0.96	2.60	< 0.001*
Child has a Secondary Diagnosis	− 0.08	0.92	< 0.001*	0.72	2.05	< 0.001*
Caregiver Involvement in Therapy	0.14	1.15	< 0.001*	− 0.39	0.68	< 0.001*
Provider Language other than English	− 0.04	0.96	0.09	− 0.25	0.78	< 0.001*
Provider is a Therapist	− 0.21	0.81	< 0.001*	− 0.35	0.70	< 0.001*
Exclusive Office-Based Care	0.58	1.78	< 0.001*	− 1.70	0.18	< 0.001*
Year of First Claim	− 0.41	0.67	< 0.001*	− 0.52	0.59	< 0.001*

*Denotes *p* < 0.05. Reference group is Profile 1 (Standard EBP Care). A positive log odds indicate a higher risk of being in a given profile relative to being to Profile 1. A negative log odds indicate a lower risk of being in a given profile relative to being in Profile 1. A relative risk ratio > 1 indicates a higher risk of being in a given profile relative to being in Profile 1. A relative risk ratio < 1 indicates lower risk of being a given profile relative to being in Profile 1

#### Profile 2: Less EBP Care

Youth’s age, language, ethnicity, primary diagnosis, secondary diagnosis, and year of first claim, caregiver involvement in psychotherapy, provider type, and service setting were significant predictors of youth’s membership in the Less EBP Care pattern. Adolescents and transition-aged youth had greater odds of receiving Less EBP Care relative to children, with effects particularly pronounced for transition-aged youth. Youth whose primary language was Spanish had greater odds of being in the Less EBP Care group relative to youth whose primary language was English. Asian American/Pacific Islander youth had greater odds of being in the Less EBP Care group relative to Hispanic youth. Youth with primary diagnoses of externalizing disorders and trauma/stress-related disorders were less likely to be in the Less EBP Care group relative to youth with primary internalizing diagnoses. Youth with a secondary diagnosis were also less likely to be in the Less EBP Care group. Higher caregiver involvement in psychotherapy was associated with greater odds of being in the Less EBP Care group, whereas having a therapist as a provider was associated with lower odds. Exclusive office-based care was associated with higher odds of being in the Less EBP Care group relative to youth receiving services in other settings. All of these effects are significant when accounting for youth’s year of first claim. Youth’s gender and provider language were not significant predictors.

#### Profile 3: Repeated/Chronic Care

Youth’s age, gender, ethnicity, primary diagnosis, secondary diagnosis, year of first claim, caregiver involvement in psychotherapy, provider type, provider language, and service setting were significant predictors of youth’s membership in the Repeated/Chronic Care pattern. Adolescents and transition-aged youth had again greater odds of being in the Repeated/Chronic Care group relative to children, with effects more pronounced for transition-aged youth. Males had greater odds of being in the Repeated/Chronic Care group relative to females. Youth who are Non-Hispanic White or from a minoritized racial/ethnic group (other than Black or Asian American/Pacific Islander) had greater odds of being in the Repeated/Chronic Care group relative to Hispanic youth. Youth with primary externalizing, trauma/stress-related, and other diagnoses (e.g., substance use) had greater odds of being in the Repeated/Chronic Care group relative to youth with primary internalizing disorders. Youth with a secondary diagnosis had greater odds of being in the Repeated/Chronic Care group and this was one of the strongest effects in the model. Youth with higher caregiver involvement in psychotherapy and who were served by a therapist who spoke a language other than English were associated with lower odds of being in the Repeated/Chronic Care group. Youth receiving exclusively office-based care had lower odds of being in the Repeated/Chronic Care group relative to youth receiving services in other settings, and this effect was large. All of these effects are significant accounting for youth’s year of first claim.

## Discussion

The present study examined characteristics of mental health service utilization among youth receiving psychotherapy and determined multiple youth-, provider-, and service-level predictors of utilization patterns within a large administrative dataset reflecting nearly six million claims over eight years in the context of a system-driven multiple EBPs implementation. Based on frequency, course, cost, and type of services, three distinct profiles of care were identified: (1) Standard EBP Care, (2) Less EBP Care, and (3) Repeated/Chronic Care. Although service receipt does not indicate level of need or clinical outcomes, understanding different profiles of care and characteristics that predict who accessed which profile of care have implications for efforts to address disparities with the goal of optimizing services for youth.

The clear majority of youth in the system (86.3%) received services designated as Standard EBP Care. Youth in this profile received psychotherapy sessions relatively consistently, both in attendance and EBP focus, over a service period that lasted under eight months. This finding may speak to the success of the system-wide implementation efforts in LACDMH over the past decade: the majority of youth receiving PEI funding received EBP psychotherapy services on a timeline congruent with the time-limited nature of many child EBPs. This finding is consistent with a recent evaluation of the PEI program in LACDMH. Ashwood et al. (2018) demonstrated that PEI services were associated with clinical improvements: of the youth who entered care with clinically significant symptoms, over half fell below the clinical significance threshold. Other studies focused on the sustainment of EBPs in LACDMH have also documented the specific implementation conditions—including the availability of EBP consultation along with therapist and program leader perceptions about EBPs (Lau et al., in press)—that have likely contributed to the prevalence of this utilization pattern.

In comparison to Reid et al. (2019), our findings as a whole are more optimistic: the majority of youth in the Reid et al. study (53%) received “Minimal” care and only 8% of youth received “Acute” care, which is comparable to the duration and number of sessions in our Standard EBP Care profile. In terms of the volume and duration of services, our finding that Standard EBP Care was delivered, on average, over 7.85 months is largely on par with the delivery time of an effectiveness trial testing EBPs in community settings, as were the number of days that passed between sessions, although the total number of sessions attended was higher in our sample (Weisz et al., 2012). Similarly, the mean number of sessions in the Standard EBP Care profile is higher than what was reported by Triplett et al. (2021), who found youth received an average of 12.4 sessions in a state-funded implementation of cognitive-behavioral therapy based on therapist report. Triplett et al. noted that the majority of youth (58.7%) in their sample continued to receive therapy after completing a course of EBP and that the majority of these continued therapy sessions included EBP elements (72.3%), more similar to the Repeated/Chronic Care pattern.

EBPs are time-limited in nature and designed to be delivered in a standard number of weekly psychotherapy sessions (e.g., TF-CBT is designed to be delivered in 12–15 sessions; Cohen & Mannarino, 2015). Although it is reassuring that youth accessed an average number of sessions ample enough to complete a full course of an EBP as designed, we must also consider the volume of sessions and the duration of the episode of care within a larger treatment literature. Studies describing mental health service dose and response are limited and inconsistent. For example, whereas one study with adults found that a minimum of four therapy sessions were necessary to achieve more than 50% reliable and clinically significant improvement (Delgadillo et al., 2014), Anderson et al. (2001) found that somewhere between 11 and 16 therapy sessions resulted in 50% clinically significant improvement. From the children’s mental health services literature, there is some evidence that the *type* of treatment moderates the dose–response relation. In a sample of children receiving usual care services, Bickman et al. (2002) found youth response was unrelated to the amount of treatment received. More recently, youth receiving cognitive-behavioral therapy in a school mental health program experienced full benefits of the treatment over the course of 14 sessions (Kirk et al., 2019). An area for future research is to connect utilization data to clinical outcomes. Further, research can dissect the Standard EBP Care pattern at a more granular level and additional correlates not examined in this study. This can help further our understanding of standard EBP practice in community mental health and differential associations with psychotherapy process, quality, engagement, and outcomes.

A portion of youth in the sample (8.5%) received a pattern of utilization labeled as Less EBP Care. This pattern is characterized by relatively consistent psychotherapy sessions across a limited time-frame (7 months), but with sessions that were not associated with the delivery of EBPs according to administrative claims. The lack of EBP sessions relative to the other utilization patterns may signal ‘lower quality’ services or implementation failure. Given the literature documenting EBPs associated with better youth outcomes (Weisz et al., 2013), this utilization pattern indicates room for improvement. There is, however, some evidence that youth in this care pattern may have different needs or may be utilizing mental health services in a different way. In particular, youth in this pattern had the highest number of crisis management claims relative to the other two care patterns, which may have reduced reliance on EBPs.

Several youth characteristics were associated with Less EBP Care. Whereas younger children were more likely to receive Standard EBP Care, adolescents and transition-aged youth were more likely to receive Less EBP Care. Youth’s age emerged as one of the strongest predictors. Research has documented a lack of sensitivity to developmental differences between children and adolescents among existing prevention and intervention programs (Malti, et al., 2016). Our findings suggest there was difficulty retaining youth of older ages in EBP care and may reflect a need to tailor existing EBPs to more effectively engage older youth and address their unique mental health service needs (Malti, et al., 2016).

Spanish-speaking youth were more likely than their English-speaking counterpart to be classified in Less EBP Care, possibly highlighting incongruence between existing EBPs with Spanish-speaking populations and/or the need to translate existing EBPs into additional languages for better service engagement. Furthermore, Asian American/Pacific Islander youth were more likely than Hispanic youth to receive Less EBP Care. This finding may be consistent with literature documenting challenges in engaging Asian American youth or families in evidence-based interventions or needed services (Guo et al., 2014; Kim et al., 2018; Kodish et al, 2020). This may point to the urgent need for culturally sensitive care and efforts to identify strategies to better align intervention with the needs of Asian American youth and families. It could also suggest the need to work closely with therapists who serve Asian American families to ensure they feel efficacious in tailoring EBPs for their clients’ needs (Lau et al., 2010). In contrast, Hispanic youth in this sample were more likely to be represented in the Standard EBP Care group. Although this finding may be evidence that the LACDMH system has adapted to address the needs of its most populous subgroup, Hispanic youth, we must also highlight that Hispanic youth still represent a sizable proportion of the other service patterns; Hispanic youth have variable experiences in EBP service use within the system. Finally, youth with externalizing and trauma/stress-related disorders were less likely to be in the Less EBP Care group. This may speak to the availability of EBPs for these specific presenting concerns within the LACDMH implementation context. For example, LACDMH furnished training and implementation supports for nine EBPs for trauma and twelve EBPs for disruptive behaviors and conduct problems, versus three EBPs for anxiety.

Several provider and service characteristics were also related to Less EBP Care. Youth who exclusively received office-based care were more likely to be in the Less EBP Care group relative to youth who received services in other settings. Coupled with the finding that youth with a secondary diagnosis were also less likely to be in the Less EBP Care group, it may be that youth with more intensive needs are more likely to receive in-home care than youth with less intensive needs. Additionally, the Less EBP Care group appear to receive a higher percentage of non-psychotherapy services (e.g., case management, evaluation and assessment) which may only be offered in clinics. Higher caregiver involvement was also associated with Less EBP Care. This finding is somewhat surprising and is difficult to reconcile with the notion of caregiver attendance in child therapy as a quality indicator of psychotherapy and a precondition for the delivery of many EBP strategies (Barnett et al., 2019; Sun et al., 2019; Wright et al., 2019). This finding may reflect a lower preponderance of caregiver-mediated EBPs for internalizing versus externalizing problems broadly and within in the LACDMH implementation specifically. Indeed, further inspection revealed a higher proportion of youth with primary internalizing disorders in this care pattern relative to the other two patterns. Thus, when caregivers are in attendance, therapists may be more likely to deliver interventions other than those structured by PEI EBPs.

The final utilization pattern, Repeated/Chronic Care (5.2%), represents repeated, costly episodes of care with a greater number of unique EBPs used. Youth in this care pattern may experience higher acuity of mental health problems and require repeated and longer-term care. Although the current study cannot adequately quantify the level of need among youth in this care pattern without clinical data, clinical comorbidity as indicated by the presence of a secondary diagnosis, was uniquely related to higher odds of receiving Repeated/Chronic care. Given the mono-problem focus of many EBPs, it is possible that youth may receive an EBP to first address a primary diagnosis, then another EBP to address the secondary diagnosis. However, it is also possible that other factors may contribute to this care pattern beyond clinical complexity or impairment. For example, youth may receive multiple episodes of care because they do not initially receive an EBP that fully meets their needs, either as a result of incomplete assessment or provider lack of training in an EBP that matches primary presenting concerns. Previous research in the LACDMH context has shown that therapists sometimes extend the use of EBPs they are trained in to alternate problem foci than originally intended (off-label use) (Kim et al., 2018; Park et al., 2018). Future studies can examine the match between EBP received and presenting concern and how (mis)match contributes to utilization patterns and outcomes. Furthermore, future work should incorporate measures of whether youth are completing a full course of an EBP within a treatment episode vs. discontinuing prematurely and thus requiring return to care. Notably, this care pattern is associated with significant costs that are four to six times the average cost of the other two patterns. From a financial standpoint, there is an opportunity to optimize treatment response to reduce costs to families and systems.

In addition to comorbidity, several other youth characteristics predicted Repeated/Chronic Care. Adolescents and transition-aged youth were also more likely to receive Repeated/Chronic Care. This may reflect the importance of intervening earlier in development before mental health problems become more intractable (McGorry & Mei, 2020). Alternately, it may suggest that PEI EBPs—meant to address mental health conditions early in their developmental course—may not address the needs for older youth as well such that they require repeated care. Youth who are Non-Hispanic White and youth from another minoritized racial/ethnic group were more likely to be in the Repeated/Chronic Care pattern than Hispanic youth. This requires further research to identify factors that explain racial differences in repeated care. As discussed previously, this may provide further evidence that the LACDMH system has adapted to the needs of Hispanic youth, or there may be unknown differences in characteristics of groups served in LACDMH that drive these apparent inequities (e.g., other service system involvement, initial severity). Compared to youth with internalizing disorders, youth with externalizing and trauma/stress-related disorders were more likely to receive Repeated/Chronic Care. Paired with the finding that caregiver involvement was associated with a lower likelihood of being in this care pattern, this may speak to the challenge and importance of involving caregivers in care, particularly for conduct and trauma-related problems for which caregiver-mediated interventions are front line (Barnett et al., 2019).

Youth who were served by multilingual providers were less likely to be classified in the Repeated/Chronic Care pattern, suggesting the importance of a multicultural and multilingual workforce. Youth who exclusively received office-based care were less likely to be in the Repeated/Chronic Care pattern. This finding may reflect the discontinuous nature of school and home-based care. For example, therapy provided in schools may be disrupted during breaks in the academic calendar. Alternatively, youth in the Repeated/Chronic Care pattern may be more functionally impaired and require more intensive home-based services. It is also possible that youth in this care pattern may experience more barriers to attend office-based therapy such as transportation and childcare needs. Future research would benefit from examining individual, familial, and neighborhood level social determinants of care that may also impact mental health care utilization patterns.

Although this study’s focus on PEI outpatient services has precluded any group comparisons at higher levels of care, these findings highlight the persistent need for mental health systems to promote equitable care. Ongoing advocacy efforts (APA, 2019; Butler & Rodgers, 2019) emphasize the importance of providing services that (1) address contextual factors unique to youth with minoritized identities (e.g., experiences of discrimination, stigma-related barriers, diversifying the mental health workforce), (2) support families (e.g., accessible and culturally competent parent education programs), and (3) buffer against socioeconomic disadvantage (e.g., adjunct early childhood programs and housing programs).

Notably, we did not identify a pattern resembling a “drop out” group that is often documented in the literature or what Reid et al., (2011, 2019) characterized as “Minimal Care.” This may speak to the success of the LACDMH context adapting to and effectively engaging with its client population. It is also plausible that children who are found eligible for PEI care in the current context differ from children served in other outpatient public sectors where higher dropout rates have been documented. In the current sample, there were nevertheless 5.8% of youth who only received one single psychotherapy session, although combined with other service use indicators, there was not a coherent profile that corresponded to a discrete “drop out” group.

### Implications

Taken together, the utilization profiles may point to the need for tailored implementation strategies and innovative models of service delivery to promote accessible, effective, and efficient care in systems implementing multiple EBPs. Findings related to the Less EBP group reveal problems with youth not receiving EBP care even in contexts resourced for implementation. More research is needed to identify mechanism(s) underlying why some youth received EBP care while some did not within a single system of care and whether tailored implementation supports may be required for providers to sustain EBP delivery. The Repeated/Chronic pattern, where youth are receiving high levels of costly care for long periods of time, highlights the importance of optimizing treatment. Youth with high-cost utilization tend to be diagnostically complex presenting with multiple comorbidities (Dickson et al., 2019). It may be expected that some portion of youth treated first within a brief, limited, early intervention approach would be subsequently identified as having more persistent service needs. The dissemination of transdiagnostic approaches that target underlying mechanisms common across child mental health problems (e.g., Unified Protocol, the FIRST treatment program) as opposed to problem-specific EBP protocols may drive more efficient care for youth with comorbidities. Measurement-based care approaches may help providers identify when clients with different diagnostic profiles, developmental needs, and social determinants of health have benefitted sufficiently from particular EBPs, redirecting care resources to more clients in need. Furthermore, stepped-care models have the potential to reduce cost and improve efficiency by first offering low-intensity interventions and only stepping up to a higher intensity intervention for youth who are not responding to treatment (Salloum et al., 2014). For example, Salloum et al. (2014) found that their stepped care TF-CBT intervention not only resulted in comparable treatment benefit compared to standard TF-CBT, it also reduced costs to families by 51.3%. This model may more efficiently serve some youth who fell in the Repeated/Chronic group by delivering low-intensity evidence-based interventions, and stepping up to weekly therapy sessions only for youth who require more intensive care.

### Limitations

This study has a number of limitations to consider. Although we described service utilization profiles based on extant literature, we did not include clinical outcome data in our analyses. It is possible that youth in the Less EBP or Repeated/Chronic utilization patterns may nonetheless experience successful treatment response. These data are also limited to the PEI Program in Los Angeles County, representing a minority of mental health services provided; PEI funding represents 19% of Mental Health Services Act allocations in LACDMH. As described earlier, PEI guidelines include a number of restrictions that make it unique; for example, when billing PEI, providers are mandated to deliver from an approved menu of EBPs. Similarly, although EBPs may be delivered in different settings, PEI is intended for outpatient services for youth in the early stage of their course of illness. This reflects a relatively low ceiling to the acuity of the youth included in this sample. Furthermore, we do not have data regarding youth’s receipt of services outside of PEI if PEI did not meet their needs or if they were deemed ineligible to receive PEI care at some point. Many additional factors that likely influence service utilization were not addressed in this study, such as social determinants of health including access issues (e.g., transportation) and poverty-related barriers to care (e.g., caregivers requiring childcare). Future studies may also explore the influence of systemic and policy factors that may influence utilization patterns over time.

## Conclusion

Using latent profile analyses, this study identified three distinct patterns of outpatient mental health service utilization in a community multiple EBPs mental health system. Within this context, the majority of youth received services aligned with many child EBP protocols, speaking to the success of the system-wide implementation of EBPs. However, a portion (13.7%) of youth received care patterns designated as Less EBP or Repeated/Chronic Care. Findings highlight opportunities to improve and optimize services, particularly for youth who are adolescents or transition-aged, Asian-American/Pacific Islander, Spanish-speaking, or presenting with comorbidities.

## Data Availability

Data analyzed in this study are owned by the Los Angeles Department of Mental Health (LACDMH), and as such, are not publicly available. Data may be available from LACDMH upon reasonable request and with permission.
